# Vascular Endothelium Derived Endothelin-1 Is Required for Normal Heart Function after Chronic Pressure Overload in Mice

**DOI:** 10.1371/journal.pone.0088730

**Published:** 2014-02-11

**Authors:** Susi Heiden, Nicolas Vignon-Zellweger, Shigeru Masuda, Keiko Yagi, Kazuhiko Nakayama, Masashi Yanagisawa, Noriaki Emoto

**Affiliations:** 1 Department of Clinical Pharmacy, Kobe Pharmaceutical University, Kobe, Japan; 2 Division of Cardiovascular Medicine, Department of Internal Medicine, Kobe University Graduate School of Medicine, Kobe, Japan; 3 University of Texas Southwestern Medical Center, Howard Hughes Medical Institute, Dallas, United States of America; Rutgers New Jersey Medical School, United States of America

## Abstract

**Background:**

Endothelin-1 participates in the pathophysiology of heart failure. The reasons for the lack of beneficial effect of endothelin antagonists in heart failure patients remain however speculative. The anti-apoptotic properties of ET-1 on cardiomyocytes could be a reasonable explanation. We therefore hypothesized that blocking the pro-apoptotic TNF-α pathway using pentoxifylline could prevent the deleterious effect of the lack of ET-1 in a model for heart failure.

**Methods:**

We performed transaortic constriction (TAC) in vascular endothelial cells specific ET-1 deficient (VEETKO) and wild type (WT) mice (n = 5–9) and treated them with pentoxifylline for twelve weeks.

**Results:**

TAC induced a cardiac hypertrophy in VEETKO and WT mice but a reduction of fractional shortening could be detected by echocardiography in VEETKO mice only. Cardiomyocyte diameter was significantly increased by TAC in VEETKO mice only. Pentoxifylline treatment prevented cardiac hypertrophy and reduction of fractional shortening in VEETKO mice but decreased fractional shortening in WT mice. Collagen deposition and number of apoptotic cells remained stable between the groups as did TNF-α, caspase-3 and caspase-8 messenger RNA expression levels. TAC surgery enhanced ANP, BNP and bcl2 expression. Pentoxifylline treatment reduced expression levels of BNP, bcl2 and bax.

**Conclusions:**

Lack of endothelial ET-1 worsened the impact of TAC-induced pressure overload on cardiac function, indicating the crucial role of ET-1 for normal cardiac function under stress. Moreover, we put in light a TNF-α-independent beneficial effect of pentoxifylline in the VEETKO mice suggesting a therapeutic potential for pentoxifylline in a subpopulation of heart failure patients at higher risk.

## Introduction

Vascular endothelial cells are the main source of the vasoactive peptide endothelin-1 (ET-1) but cardiomyocytes, endocardial cells, and cardiofibroblasts produce ET-1 as well as its both receptors ET_A_ and ET_B_ [1]. The involvement of the endothelin system in the pathophysiology of congestive heart failure has been recognized early after the discovery of ET-1. The circulating and tissue ET-1 levels increase in the failing heart and correlate with the severity of the disease in patients and animal models [2], [3]. Hypertrophic, fibrotic, pro-inflammatory and inotropic effects of ET-1 contribute to the development of heart failure [4]. Most of these deleterious effects are attributed to the activation of ET_A_ receptors.

Treatment with selective ET_A_ as well as dual ET_A_/ET_B_ antagonists demonstrated beneficial effects in several animal models of acute and chronic heart failure [5]–[7]. Both ETA and ETB receptors might play additive roles in the pathological cardiac remodelling [5]. However, trials of endothelin receptor antagonists have not shown the expected clinical benefits [8], [9]. Several reasons have been discussed which could account for this disappointing outcome. Among others, the application of inadequate animal models for preclinical studies, the difficulty to show additional benefit in already medicated patients or incorrect dose or timing of treatment [10].

Despite its adverse effect on the heart, overexpression of ET-1 in mice can prevent diastolic dysfunction in eNOS deficient mice [11]. Moreover, anti-apoptotic properties of ET-1 on cardiomyocytes have been observed in vitro [12], [13] and in vivo in mice with cardiomyocyte specific ET-1 deletion [14]. These mice developed dilated cardiomyopathy with impairment of heart function as a response to stress. It was presumed, that ET-1 reduced the pro-apoptotic TNF-α signalling.

We performed transaortic constriction in ET-1 deficient mice to further examine the impact of ET-1 on the heart subjected to increased afterload. Treatment with pentoxifylline (PTX) was aimed to reduce TNF-α synthesis and by doing so to demonstrate the influence of ET-1 on the TNF-α signalling.

## Methods

### Experimental design

We used non-ovariectomised female mice with vascular endothelium specific ET-1 deficiency (ET-1^flox/flox^, Cre recombinase positive: VEETKO) and their wild type littermates (ET-1^flox/flox^, Cre recombinase negative: WT) [15]. The mice were housed in a temperature controlled environment (22–24°C) with a 12-hour light and dark cycle and had free access to water and a standard chow. A total of 85 mice were used for this experiment. The final number of mice per group varied from five to nine depending on the group. At the age of eight weeks, the mice were randomized and either underwent transverse aortic constriction (TAC) using a 26Gy diameter needle or sham surgery. The operation was performed under anaesthesia by isoflurane. To reduce suffering, the mice received two injections of buprenorphine (0.1 mg/kg, Lepetan, Otsuka, Japan) right after and 12 hours after the surgery. Treatment with pentoxifylline (PTX) started one week after surgery. PTX was administered via drinking water (0.5 g/L). The dose received by the mice was thus on average 90 mg/kg/day. Bottles were protected from light. Untreated mice received normal water. Twelve weeks after operation, blood pressure and cardiac function were measured. The mice were then sacrificed by cervical dislocation. Hearts were withdrawn and washed in cold phosphate buffered saline; one half was snap-frozen in liquid nitrogen for protein and RNA extraction and one half was embedded in paraffin for histological investigation.

### Ethics Statement

All animal experimental protocols were conducted in accordance with the Guidelines for Animal Experiments at Kobe Pharmaceutical University and were approved by The Animal Research and Ethics Committee of Kobe Pharmaceutical University, Kobe, Japan. Adequate anesthetics and analgesics were used to reduce pain in the mice during and after surgery (see “Experimental design” section).

### Blood pressure measurement

Blood pressure and heart rate were measured in awake mice by the tail-cuff method (Softron BP-98A, Softron, Tokyo, Japan) between 9 a.m. and noon. Mice were trained to the procedure on the first day and measurements were recorded on the second day. An average of ten consecutive measurements was used.

### Echocardiography

Left ventricular end-diastolic (EDD) and end-systolic dimension (ESD) were measured by echocardiography (Envision, Philips). Two-dimensional parasternal short-axis images were obtained, and targeted M-mode tracings at the level of the papillary muscles were recorded. Fractional shortening (FS) was calculated using the formula (EDD-ESD)/EDDx100. Examinations were performed within ten minutes of light isoflurane anaesthesia.

### Histology

Paraffin embedded heart were cut into 3 µm and 1 µm sections and submitted to Sirius-red and Hematoxylin-eosin staining. Interstitial fibrosis and myocyte diameter was quantified using a computer-aided image analysis system.

### Real-time PCR

Total RNA was extracted from cardiac tissue using Trizol reagent (15596-026, Invitrogen, Japan) following manufacturer's protocol. Complementary DNA was obtained by reverse transcription using oligo-dT primers and the ReverTra Ace kit (FSQ-101, Toyobo, Osaka, Japan). Real time PCR was performed using the Thunderbird SYBR qPCR mix (QPS-201,Toyobo, Osaka, Japan) on a Rotor-Gene Q thermocycler (Qiagen, Tokyo, Japan). The primers for the PCR reaction are presented in the table 1. Condition of the PCR was: initial denaturation at 94°C for 1 min followed by 40 cycles of annealing at 60°C for 1 min and denaturation at 94°C for 15 s. Relative gene expression was calculated by the ΔΔC_T_ method using actin expression as reference.

**Table 1:** List of real-time PCR primer sequences.

Gene	Primer sequences	amplicon size (bp)
ET-1	TGAGTTCCATTTGCAACCGAGT	152
	CTGAGTTCGGCTCCCAAGAC	
TNFα	CATCTTCTCAAAATTCGAGTGACAA	175
	TGGGAGTAGACAAGGTACAACCC	
Bax	CAGGATGCGTCCACCAAGAA	165
	GTTGAAGTTGCCATCAGCAAACA	
Bcl2	GTGTTCCATGCACCAAGTCCA	127
	AGGTACAGGCATTGCCGCATA	
Caspase 3	CGTGGTTCATCCAGTCCCTTT	102
	ATTCCGTTGCCACCTTCCT	
Caspase 8	ACAATGCCCAGATTTCTCCCTAC	175
	CAAAAATTTCAAGCAGGCTCA	
Actin	CATCCGTAAAGACCTCTATGCCAAC	171
	ATGGAGCCACCGATCCACA	
ANP	TGACAGGATTGGAGCCCAGAG	138
	AGCTGCGTGACACACCACAAG	
BNP	ATCGGATCCGTCAGTCGTTTG	94
	CCAGGCAGAGTCAGAAACTGGAG	

### Statistical analysis

Values were presented as mean±sem. Differences between groups were analysed using a Student's T-test for independent samples on the software SPSS. A p value less than 0.05 indicated a significant difference.

## Results

### Blood pressure

Neither genotype nor TAC surgery had any influence on systolic and diastolic blood pressure. In sham-operated WT mice but not in VEETKO, PTX treatment increased SBP. SBP was thus lower in VEETKO mice compared to WT in sham-operated mice receiving PTX. In mice with TAC, SBP and DBP remained stable throughout the experiment regardless of treatment received by the mice (table 2).

**Table 2:** Cardiac morphology and function in sham-operated and TAC WT and VEETKO mice twelve weeks after operation, receiving PTX or not.

	Sham-operated mice				TAC mice			
	control		PTX treated		control		PTX treated	
	WT	VEETKO	WT	VEETKO	WT	VEETKO	WT	VEETKO
n	5	7	5	8	5	9	6	9
Body weight (g)	25,3±2,3	28,5±2,1	24,7±0,8	29,4±1,6	26,1±2,4	27,4±1,3	26,7±2,3	26,3±1,2
Heart weight/body weight (mg/g)	0,43±0,03	0,43±0,01	0,43±0,01	0,41±0,01	0,54±0,03*	0,60±0,04*	0,55±0,02*	0,50±0,01*,‡
Heart weight/tibia length (mg/mm)	0,62±0,04	0,65±0,06	0,58±0,03	0,61±0,01	0,85±0,08*	0,86±0,07*	0,77±0,03*	0,73±0,04*
Cardiomyocytes diameter (µm)	13,5±0,4	13,5±0,8	13,0±0,6	13,5±0,2	15,7±1,1	16,5±0,5*	15,2±1,0	14,6±0,8
Heart rate (bpm)	624±14	657±10	657±15	646±23	643±3	648±12	688±22	662±13
Systolic blood pressure (mmHg)	108±3	112±2	122±2||	113±2	113±6	104±3	111±5	106±3
Left ventricular end systolic dimension (mm)	1,33±0,20	1,25±0,17	1,33±0,19	1,44±0,15	1,19±0,15	1,63±0,09*,†	1,56±0,17	1,19±0,13‡
Left ventricular end diastolic dimension (mm)	2,93±0,08	2,67±0,21	2,87±0,11	2,91±0,19	2,73±0,26	3,14±0,11*	2,97±0,20	2,52±0,20‡
Fractional shortening (%)	52,7±4,7	54,6±2,9	51,2±3,2	50,5±3,1	56,8±2,4	48,4±1,6*,†	46,1±1,3†	53,1±3,2‡,§

### Cardiac structure and function

**Figure 1:**
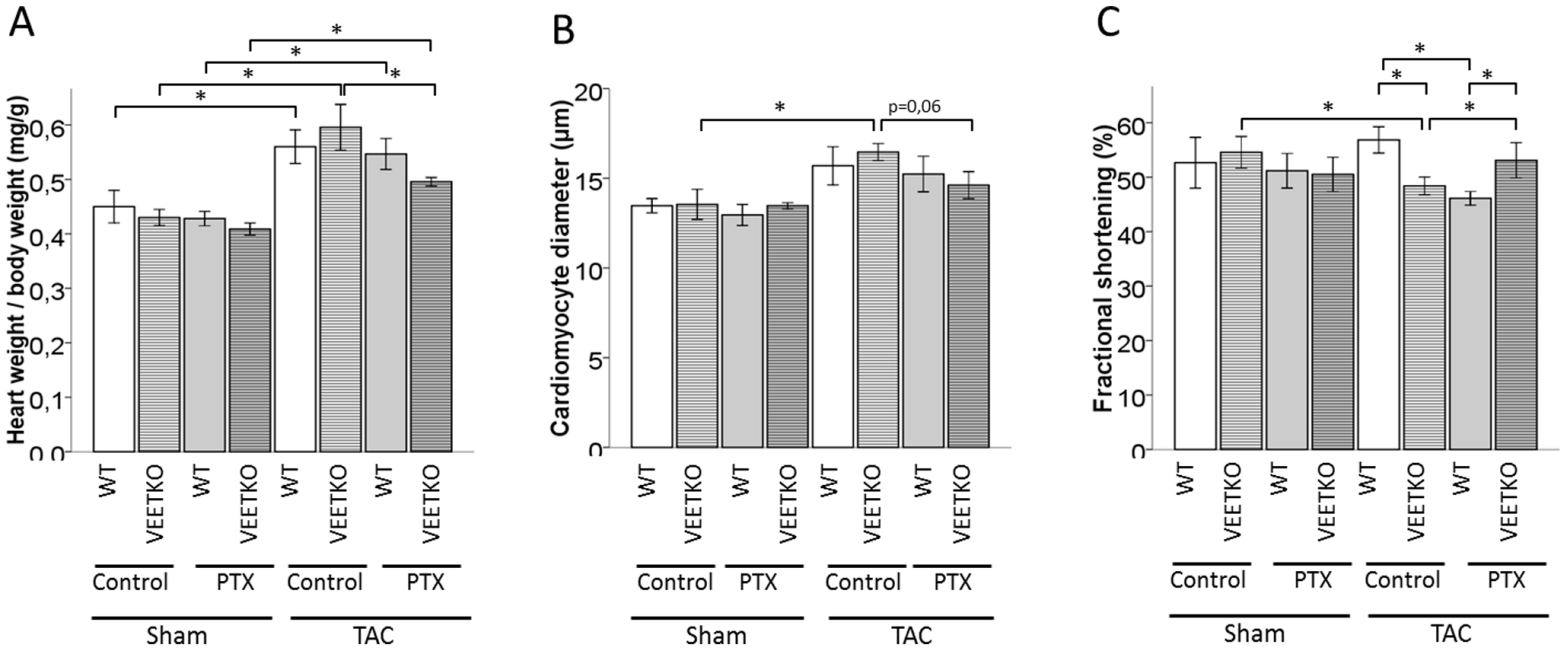
TAC-induced cardiac hypertrophy and reduction of cardiac function in VEETKO mice was prevented by PTX. (A) Heart weight to body weight ratio. (B) Cardiomyocyte diameter measured on hematoxylin-eosin stained sections. (C) Fractional shortening measured by echocardiography. Values are mean±sem, n = 6–9, Student's T-test: * p<0.05.

In sham-operated mice, neither genotype nor PTX treatment influenced heart weight to body weight ratio. TAC induced cardiac hypertrophy in WT and VEETKO mice in a similar fashion (figure 1A). PTX treatment reduced cardiac weight when normalized to body weight in VEETKO mice but not in WT (table 2). TAC induced an enlargement of cardiomyocytes in both WT and VEETKO mice but it was statistically significant in VEETKO mice only (figure 1B). PTX treatment had no statistically significant effect on myocyte diameter either in sham-operated or in TAC mice. We could however note a tendency to a reduction of cardiomyocyte diameter by PTX in TAC-VEETKO mice (p = 0.06) (figure 1B). Using echocardiography, we observed a TAC-induced enlargement of the ESD and EDD in the left ventricle of VEETKO mice but not of WT mice. ESD was thus significantly bigger in VEETKO mice with TAC compared to WT mice. PTX treatment restored ESD and EDD in VEETKO mice to the level of sham-operated mice (table 2). Fractional shortening was similar in sham-operated mice and PTX treatment had no impact in these mice. TAC surgery led to a decrease of FS in VEETKO mice but not in WT. Consequently, twelve weeks after TAC, FS was smaller in VEETKO mice than in WT. PTX treatment had opposite effects whether given to WT or VEETKO mice: PTX decreased FS in WT mice which had undergone TAC but restored FS in VEETKO mice to the level of the sham-operated mice. FS was thus higher in VEETKO mice than in WT when treated with PTX (figure 1C).

Taken together, TAC reduced cardiac function in VEETKO mice but not in WT and this decrease could be prevented by a PTX treatment.

### Histology

**Figure 2:**
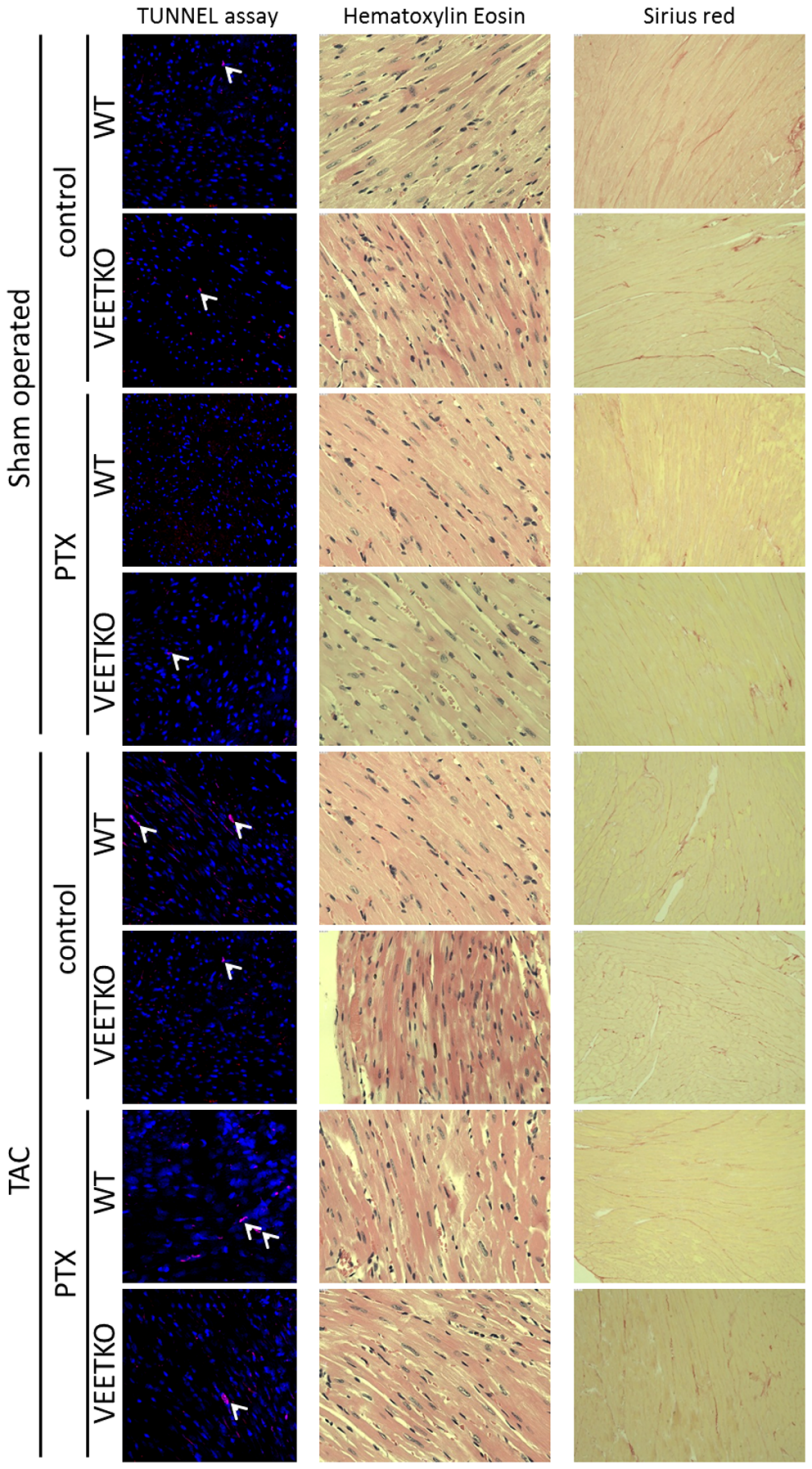
Apoptotic myocyte number and cardiac collagen deposition remained stable between the groups while TAC induced cardiomyocyte enlargement, significantly in VEETKO mice only. TUNEL staining (left panel), hematoxylin-eosin staining (central panel) and Sirius red staining (right panel) show apoptotic cells, tissue structure and collagen deposition, respectively. Quantification of cardiomyocyte diameter based on hematoxylin-eosin staining is shown in figure 1C.

No difference has been observed between the groups regarding the level of collagen deposition defined as Sirius red positive signal. The number of apoptotic cells counted after TUNEL assay was low and similar between the groups (figure 2).

### Cardiac gene expression

**Figure 3:**
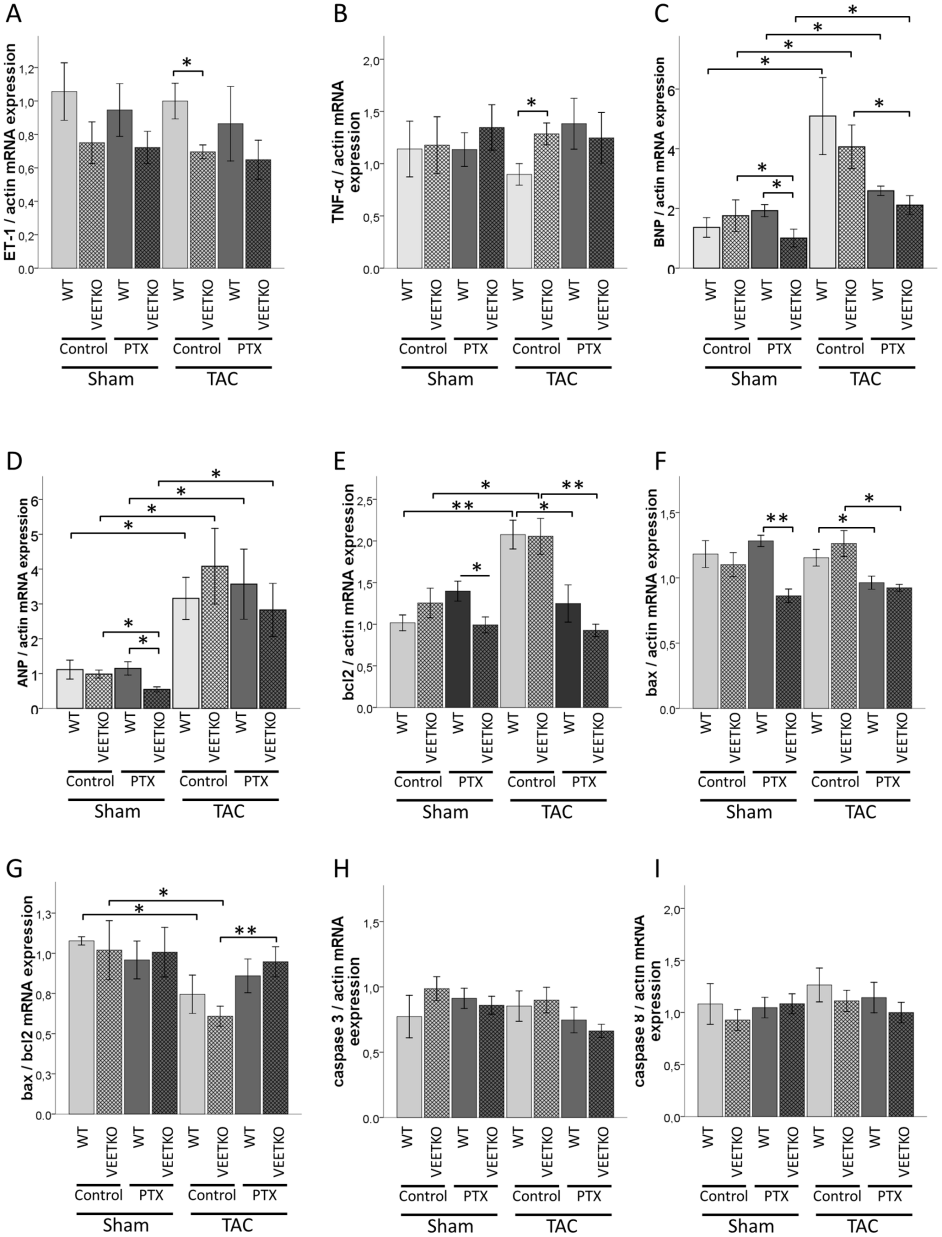
Gene expression level of (A) ET-1, (B) TNF-α, (C) ANP, (D) BNP, (E) bcl2, (F) bax,(H) caspase 3 and (I) caspase 8. Messenger RNA expression levels were determined by real time-PCR analysis and normalized to actin expression using the ΔΔC_T_ method. (G) The expression ratio bax/bcl2 was also calculated. Values are mean±sem, n = 6–9, Student's T-test: * p<0.05; ** p<0.01.

In all groups (sham- or TAC operated, treated with PTX or not), the gene expression of ET-1 was lower in VEETKO compared to WT mice. In our setting, the difference was statistically significant only in the control mice after TAC surgery (figure 3A). In these groups, the reduced ET-1 expression in VEETKO mice was accompanied by a higher TNF-α gene expression (figure 3B). In sham-operated mice, PTX treatment reduced bcl2 and bax expression in VEETKO mice when compared to WT. The TAC surgery increased the gene expression of bcl2 regardless of the genotype. This increase was reversed by the PTX treatment. In TAC mice, PTX decreased the expression of bax as well (figure 3E–F). The expression ratio bax/bcl2 was thus lower in TAC mice, and restored by PTX (significantly in the VEETKO only) (figure 3G). Like bcl2, the mRNA level of ANP and BNP was reduced in VEETKO mice treated by PTX compared to the WT. Both ANP and BNP mRNA levels were increased in all TAC mice. The gene expression of BNP was reduced by PTX treatment (significantly in VEETKO mice only) (figure 3C–D). The gene expression of caspase-3 and caspase-8 was not affected by genotype, surgery or treatment (figure 3H–I).

## Discussion

The main findings of this study are that a normal expression level of ET-1 is required to maintain cardiac function after pressure overload caused by transaortic constriction and that the adverse impact of a reduced expression of ET-1 can be prevented by a pentoxifylline treatment.

### Role of ET-1 on cardiac hypertrophy, heart function and apoptosis after TAC

ET-1 is known to have strong hypertrophic effects on the heart [7]. Thus, in mice with myocardial deletion of ET-1, the hypertrophic response to an acute hormonal stimulus is not as strong as in wild type mice [16]. In contrast, the same mice subjected to TAC develop a stronger hypertrophy than the WT mice [14]. In our experiment, the absence of vascular ET-1 had no statistical influence on the hypertrophic response to TAC measured as heart weight to body weight ratio. Based on previous studies, we are confident that the ET-1 peptide levels are significantly decreased in the myocardium of the VEETKO mice [15], [17]. Differences in terms of endothelin expression exist between sexes [11], [18] and might explain that the ET-1 levels observed in the present study differ from already published reports. A limitation to our model would be that cardiomyocytes and fibroblasts remain a significant source of ET-1 in the VEETKO mice. Nevertheless, in response to the ET-1 suppression the TAC-induced increase in cardiomyocytes diameter was statistically higher in VEETKO mice only. Albeit small, the differences between the genotypes correlated with the decrease of cardiac function. The above-cited literature, together with the data presented here, indicates that the reduction of cardiac ET-1 promotes cardiac hypertrophy in mice with increased afterload. This conclusion is supported by the work by Kedzierski et al. which showed that mice lacking the ET_A_ receptor in cardiomyocytes do not present a modified cardiac hypertrophic response to pharmacological stress [19]. In contrast, in a model of angiotensin II-induced cardiac hypertrophy, lack of endothelium-derived ET-1 prevented heart growth [20]. If angiotensin II is one the main factor in this pathological process, others like endothelin and catecholamines and products of oxidative stress are essential for the transduction of the hypertrophic signal [21]. Most importantly, the TAC model reproduces many aspects of human heart failure [22]. Finally, the discrepancies between these two animal models should be analysed in the light of the failure of clinical trials of endothelin receptor antagonists for heart failure [8], [9].

ET-1 has been held responsible for the pathophysiology of heart failure [7], before its protective role on cardiac physiology began to be revealed, in particular its anti-apoptotic properties on cardiomyocytes [12], [13]. Specifically, our study confirms the experiments using mice with myocardial deletion of ET-1 [14]. Subjected to TAC, these mice, like the VEETKO mice, suffer not only from an increased hypertrophy but from a worsening of cardiac function too, while the WT mice do not. Zhao et al. additionally observed an increase of fibrosis and a disorganization of muscle fibres, what we did not in the VEETKO mice. Their TAC model was however more severe: they used a 27-gauge syringe when we used a 26-gauge and the absence of myocardial ET-1 led to a stronger reduction of FS than the suppression of vascular endothelial ET-1 (50% compared to less than 10% in the VEETKO mice). The elevation of ESD and EDD was also more pronounced in the myocardial specific ET-1 KO mice compared to the VEETKO mice. Further, Zhao et al. observed a similar phenotype in aging myocardial specific ET-1 KO mice without TAC surgery. In these mice, they detected a higher number of apoptotic cells as well as a stronger expression of caspase-3 and caspase-8. They therefore proposed that ET-1 possessed anti-apoptotic properties on cardiomyocytes, which had been already shown in vitro [12], [13]. In parallel, several studies have shown an increase of myocardial apoptosis after TAC in mice and other experimental animals [23]–[25].

We have thus hypothesized that the reduction of cardiac function in VEETKO mice was due to the loss of anti-apoptotic properties of ET-1. In our setting, however, we could not detect changes neither in apoptotic cells number nor in caspase expression levels. This represents a major limitation of our study for which several parameters might be responsible. Apoptosis is a late event in the pathophysiology of TAC induced heart failure: Fliegner et al. (using the same protocol as ours) did not observed apoptosis nine weeks after TAC [22]. Moreover, the expression of the anti-apoptotic gene bcl2 increased in TAC mice while the expression of the pro-apoptotic bax remained stable. The expression ratio bax/bcl2 was thus decreased in TAC mice. This indicates the presence of compensatory mechanisms, which may have prevented deterioration of tissue integrity in the TAC mice. This could explain the absence of measurable apoptosis in our setting. Such an increase of bcl2 has been observed earlier in sheep subjected to aortic banding, but this increase was accompanied by an increased bax/bcl2 ratio [24]. Nevertheless, Moorjani et al. gradually increased the constriction in order to provoke LV dysfunction as soon as six weeks after operation [24]. A similar study depicts an increased and decreased expression of pro- and anti-apoptotic genes respectively after TAC as well [23]. The authors observed also an increased number of apoptotic cells which is not the case in our study. Twelve weeks after TAC, the authors observed a compensatory phase defined by cardiac hypertrophy without decrease of fractional shortening like we did (at least in the WT mice). After 24 weeks, the further increase of both the bax/bcl2 ratio and the apoptosis rate correlated with the deterioration of cardiac function (reduction of FS) [23]. Once again, our TAC model might be less severe and this may account for the absence of apoptosis. The low impact of TAC might be explained by the use of female mice, which are protected from TAC induced cardiac injury [22] compared to males [26], [27] using the same 26-gauge needle for constriction. Further, the VEETKO mice and their littermates are small compared to mice on another genetic background and we might have underestimated that the constriction of the aorta might be less on small mice. The assumption that our set-up is a model for moderate heart failure is supported by the fact that TNF-α levels remained stable in TAC mice. The level of inflammatory mediators correlates namely closely with the severity of heart failure [28]. Given that the expression of cardiac bcl2 and bax did not depend on the presence of vascular ET-1, we propose that the protective effect of ET-1 on cardiac function did not rely on a reduction of the mitochondrial apoptotic pathway. The role of ET-1 on bcl2 and bax is still disputed: on one hand, the anti-apoptotic effect of ET-1 on cardiomyocytes has been revealed in particular through its ability to increase bcl2 expression [12], on the other hand an in vitro study demonstrated that ET-1 has no influence on bax and bcl2 expression in cardiomyocytes [29].

Notably, the effects observed were independent of systemic blood pressure changes. Even though previous investigations of the VEETKO mice have revealed a blood pressure lower than in the WT, we were unable to confirm this. The endothelin system is known to participate in the sex-related differences in blood pressure control [30], [31]. The fact that we used female mice might explain the discrepancy with previous reports.

### Effect of PTX on cardiac function after TAC

Importantly, the deleterious effect of the absence of vascular ET-1 on myocardial hypertrophy and function could be prevented by PTX: fractional shortening was increased, heart weight was reduced and myocyte diameter (albeit not significantly) as well. Except from a small increase of blood pressure in the sham WT mice, for which the reasons are unknown, the effects of PTX were blood pressure independent. While some studies did not reveal improvement of cardiac structure and function in heart failure patient with PTX treatment [32] some did show a reduction of LV dimension and amelioration of cardiac function [33]–[36]. One of the commonly observed mechanisms of action of PTX is to reduce TNF-α expression. However, we haven't observed any changes in TNF-α expression after PTX treatment though. The influence of PTX on TNF-α is not clear. While some studies show a reduction in TNF-α expression by PTX [36], some failed to do so [32], [33]. Moreover, direct anti TNF-α therapies using specific antibodies did not ameliorate outcome in heart failure patients (e.g. infliximab: ATTACH trial [37], or eternecept: RENAISSANCE, RECOVER and RENEWAL trials [38]), while PTX treatment can benefit patients in the absence of a reduction of TNF-α levels [33]. The advantages of PTX versus pure anti TNF-α drugs may be that PTX slightly modulates the levels of TNF-α without blocking its cardio-protective properties [39]. A salutary effect of PTX on cardiac function without significant reduction of TNF-α level is therefore not unanticipated. Given that TNF-α mRNA expression was not changed by PTX in VEETKO mice, we can speculate on the reasons why PTX ameliorates cardiac function in these mice. Some TNF-α-independent effects of PTX are to be considered. One of them could be the anti-apoptotic effects of PTX. Even though we could not detect significant changes in the number of apoptotic cells, we have observed that PTX treatment influenced the level of expression of key proteins for the mitochondrial apoptotic pathway, bcl2 and bax. The anti-apoptotic effects of PTX and particularly its ability to regulate bcl2 and bax expression have been put in light earlier [40], [41]. Thus, the fact that PTX modified the level of expression of genes involved in apoptosis in the absence of change in TNF-α expression supports the assumption that PTX may be beneficial due to a TNF-α-independent antiapoptotic effect [42].

The changes in bax and bcl2 expression must be interpreted carefully because there were independent of the genotypes and thus did not correlate with the changes in cardiac function. The PTX-induced increase of the bax/bcl2 ratio in TAC-VEETKO mice was in contradiction with the improved cardiac function. On the other hand, PTX restored this parameter to the level of the sham-operated mice, which can be seen as a beneficial effect. Beside its anti-apoptotic effects, PTX has been shown to induce apoptosis in certain conditions [43], e.g. by increasing bax expression in a greater extent than bcl2 in tumour cells [44]. The impact of PTX on apoptosis may be complex and more detailed investigation would be needed to clarify it in the present study.

Finally, PTX treatment in the TAC mice induced a reduction of the expression of cardiac BNP as well, which is in line with a previous report [36] and can be considered as an improvement. Importantly, the restoration of BNP expression level and bax/bcl2 ratio was significant in VEETKO mice only underlining that PTX had differential impacts on both genotypes.

We thus conclude that PTX prevents TAC-induced cardiac dysfunction and hypertrophy in mice with reduced ET-1 expression.

### Discrepancy between PTX effect in WT and VEETKO mice

In contrast to its positive impact in mice with reduced endogenous endothelin-1, PTX had a deleterious effect on cardiac function in the mice with normal level of ET-1. A clinical study have shown that PTX is effective only in a sub-population of heart failure patients, which can be identified by an elevated serum concentration of inflammatory markers [35]. Similarly, we have observed that PTX was efficient only in a population which we can consider at higher risk: the VEETKO mice, which showed a higher expression level of TNF-α than the WT mice twelve weeks after TAC.

Moreover, a reason for the opposite effects of PTX on cardiac function in VEETKO and WT mice may lie on the complex pharmacology of PTX: PTX is metabolized in several active compounds. In WT mice, TAC induced solely cardiac hypertrophy while an additional reduction of FS was observed in VEETKO mice, which can be considered as a worsening of the condition. The pharmacokinetics of PTX and particularly the relative concentration of its metabolites is not the same whether given to healthy humans, patients with moderate or severe heart failure [45], [46]. Since PTX and its metabolites show different molecular actions [47], the possible differences in metabolite concentration between WT and VEETKO mice may explain the different consequences of PTX treatment.

### Conclusions

Firstly, the present study confirms the essential role of ET-1 for normal cardiac function after chronic overload and participates in explaining the negative results of endothelin antagonists in heart failure trials. Secondly, our results indicate that PTX prevents cardiac failure in mice with reduced ET-1 expression. In the absence of large scale clinical trial of PTX on heart failure, it is still difficult to conclude on its therapeutic potential. Thirdly, we have shown that PTX may have opposite effects on cardiac function depending on the pathophysiological situation. Further studies should be therefore carefully designed.
