# Correction: Vascular Endothelium Derived Endothelin-1 Is Required for Normal Heart Function after Chronic Pressure Overload in Mice

**DOI:** 10.1371/journal.pone.0099473

**Published:** 2014-06-04

**Authors:** The PLOS ONE Staff

The footnotes are missing from Table 2. Please see the complete, corrected Table 2 here.

**Table 2:** Cardiac morphology and function in sham-operated and TAC WT and VEETKO mice twelve weeks after operation, receiving PTX or not.

	Sham-operated mice				TAC mice			
	control		PTX treated		control		PTX treated	
	WT	VEETKO	WT	VEETKO	WT	VEETKO	WT	VEETKO
n	5	7	5	8	5	9	6	9
Body weight (g)	25,3±2,3	28,5±2,1	24,7±0,8	29,4±1,6	26,1±2,4	27,4±1,3	26,7±2,3	26,3±1,2
Heart weight / body weight (mg/g)	0,43±0,03	0,43±0,01	0,43±0,01	0,41±0,01	0,54±0,03*	0,60±0,04*	0,55±0,02*	0,50±0,01*,‡
Heart weight / tibia length (mg/mm)	0,62±0,04	0,65±0,06	0,58±0,03	0,61±0,01	0,85±0,08*	0,86±0,07*	0,77±0,03*	0,73±0,04*
Cardiomyocytes diameter (ìm)	13,5±0,4	13,5±0,8	13,0±0,6	13,5±0,2	15,7±1,1	16,5±0,5*	15,2±1,0	14,6±0,8
Heart rate (bpm)	624±14	657±10	657±15	646±23	643±3	648±12	688±22	662±13
Systolic blood pressure (mmHg)	108±3	112±2	122±2||	113±2	113±6	104±3	111±5	106±3
Left ventricular end systolic dimension (mm)	1,33±0,20	1,25±0,17	1,33±0,19	1,44±0,15	1,19±0,15	1,63±0,09*,†	1,56±0,17	1,19±0,13‡
Left ventricular end diastolic dimension (mm)	2,93±0,08	2,67±0,21	2,87±0,11	2,91±0,19	2,73±0,26	3,14±0,11*	2,97±0,20	2,52±0,20‡
Fractional shortening (%)	52,7±4,7	54,6±2,9	51,2±3,2	50,5±3,1	56,8±2,4	48,4±1,6*,†	46,1±1,3†	53,1±3,2‡,§

Table 2: Cardiac morphology and function in sham-operated and TAC WT and VEETKO mice twelve weeks after operation, receiving PTX or not. * p<0.05 vs. sham-operated same genotype, † p<0.05 vs. control TAC-WT, ‡p<0.05 vs. control TAC-VEETKO, § p<0.05 vs. PTX TAC-WT, || p<0.05 vs. control sham-operated WT. (WT: wild type, VEETKO: vascular endithelial endothelin-1 knock-out, TAC: transaortic constriction, PTX: pentoxifylline).

## References

[1] Heiden S, Vignon-Zellweger N, Masuda S, Yagi K, Nakayama K, et al (2014) Vascular Endothelium Derived Endothelin-1 Is Required for Normal Heart Function after Chronic Pressure Overload in Mice. PLoS ONE 9(2): e88730 doi:10.1371/journal.pone.0088730 24523936 10.1371/journal.pone.0088730 PMC3921186

